# Cases in the Medical Wards of Mercy Hospital

**Published:** 1871-10

**Authors:** 


### CLINICAL REPORTS.

---

### CASES IN THE MEDICAL WARDS OF MERCY HOSPITAL.

---

Service of PROF. N. S. DAVIS. Reported by B.

---

Case. I. Progressive Locomotor Ataxy.—Progressive Locomotor Ataxy was imperfectly recognized and described by old writers as tabes dorsalis, and was classed with paralysis. But the disease was not well understood until a few years ago, when Duchenne of Bologne investigated it thoroughly. The prominent feature is a loss of power to coördinate the muscular movements. No muscles fail to contract under the stimulus of the will as in paralysis, and the movements are made promptly, unlike the slow and imperfect motions of a paralytic who has partially recovered. But the patient cannot perfectly execute a movement that requires the action of several muscles at once, especially in the lower extremities. In attempting to walk he starts off briskly, but staggers like an intoxicated man, and steadies himself by surrounding objects; frequently he is driven into a run to preserve his balance. The patient says he "has not much strength," but in reply to questions, he says he is not tired by exertion; in fact, he might walk for miles without special weariness.

The man attributes his condition to his exposure at the time of the fire, but his friends have noticed for a year a growing unsteadiness in his gait, especially an awkwardness and difficulty in mounting steps. Among the earliest indications are alterations of sensibility in the lower extremities. Some hyperæsthesia and pain; often there is anæsthesia, especially in the soles of the feet, so the patient is not certain when he touches the ground. Soon the friends will notice that he walks strangely, though the sufferer himself thinks they are mistaken. There is generally disturbance of the special senses, particularly sight and hearing: this patient has an indescribable sensation in the ears which he attributes to exposure.

On examination of the spinal cord under the microscope, it is found that there is atrophy of the proper nerve structure, and an increase of fibrous or connective tissue. Only a small amount of nerve cells and fibres are found in the posterior roots of many spinal nerves and in some cranial nerves. Whether this atrophy results from some impairment of vital affinity or from some deficiency in the blood, is still uncertain.

*Treatment*:—It is desirable to do something to promote nutrition if possible. Habits of life must be carefully regulated, and any exercise which would exhaust energy must be prevented. Gentle currents of electricity, and sometimes friction are to be applied along the spine. For medicine, the best results are obtained from the Hypophosphites with an excess of phosphorous acid; with minute doses of strychnia, if there is constipation. Under such treatment the disease has been kept at bay in another patient for six years. Six or seven years is the average duration of the disease. It almost always terminates fatally sooner or later.

---

CASE II. INTERMITTENT FEVER, WITH PULMONARY COMPLICATIONS.—This young man was attacked by chills and fever five weeks ago, and in the course of two weeks under his physician's treatment, the chills disappeared and the general fever was cured. But since that he has been pale, coughs and sweats considerably at night. These unpleasant symptoms not unfrequently follow typhoid and intermittent fevers which have impoverished the blood and led to some exudation into the tissues of the lungs. This exudation, if not absorbed may degenerate, constituting a condition which Niemeyer calls caseous infiltration. The patient convalescing from the intermittent is nevertheless languid, short of breath, coughs, especially in the evening and morning, has quickness of pulse and heat, generally with night sweats. Unless prompt treatment is resorted to, the infiltration progresses to suppuration with fatal exhaustion in two or three months, constituting quick consumption.

In this case auscultation reveals increased density in the right lung, especially at the base, which is scarcely at all inflated except by forced inspiration. This density is due to exudation brought on by local congestion with the impoverished condition of the patient's blood. Tonics are evidently indicated, and likewise anodynes to allay irritation in the lungs. He was directed to use twice a day the muriate of ammonia mixture, previously described. Besides the anodyne influence of the mixture, the muriate of ammonia as an alterative promotes absorption. Of the many tonics which might be used the extract of malt and comp. syrup of the Hypophosphites taken at each meal constitute one of the best. Many would order cod liver oil which, however, is apt to disagree with the stomach and so impairs digestion. Syrup of Iodide of Iron, 20 drops after each meal, was directed for this patient. This gives the alterative influence of iodine with tonic properties ot the iron. In tuberculosis, also, when iron is to be used the iodide is the best form. According to the reports of the Brompton Hospital, devoted to the treatment of consumptives, this agent was represented as particularly favorable in the suppurative stage with tendency to night sweats.

---

Case III. Chronic Ague.—Attention is called to the pale and rather sallow complexion, the empty veins and bloodless appearance of a patient who has entered the Hospital after being troubled with ague more or less for four months. Malaria diminishes the amount of red corpuscles, and in extreme cases the thining of the blood produces a tendency to anasarca. By examining the blood of patients who had suffered from malaria the professor had found the red corpuscles reduced from the normal 127 in 1000 to 50 in 1000. This is a regular concomitant of intermittent, and forms a marked contrast with typhoid fever. It is uncertain whether the red corpuscles are destroyed by the direct action of the malaria or the development arrested by some change in the organs by which they are matured. But in treatment the fact is to be remembered, especially in chronic cases. The physician who is called to stop the paroxysms of intermittent frequently does nothing further, and there is a relapse with which the patient is dissatisfied, or a new disease may arise from the condition in which the intermittent leaves the patient. Intermittents can be easily interrupted, but the disease is not cured by stopping the paroxysms.

Treatment should be adopted at once to restore the blood from its pathological changes. This may be well accomplished by the use of the extract of Cornus Florida or black ash, with iron and nux vom., as in the following formulæ:

| | | |
|---|---|---|
| ℞ | Ext. Hyosciamus, | 40 grs. |
| | Ext. Cornus Florida, | 80 grs. |
| | Citrate of Iron, | 80 grs. |
| | Ext. Nux. Vom., | 20 grs. |
| M. | | |

Divide into 40 pills. Take one pill before each meal time, until the blood regains the normal proportions of its red corpuscles, and the patient's strength is restored. This patient still having slight paroxysms of intermittent character with looseness of the bowels, was directed to take Sulph. Quinia 3 grs. and Sulph. Morph. 1-4 gr. each morning and noon in addition to the use of the above pills.

---

CASE IV. DIABETES MELLITUS.—This patient for several months has had a disease, the beginning of which is very insidious. He found himself despondent, troubled with a little indigestion, and dull pain in the loins, and he had to urinate rather often. No severe symptoms appeared, and nine of ten patients at that stage imagine themselves "bilious." The appetite for food and especially for water gradually increased, and a large amount of water was passed. The skin was dry and rough. The hands are a little shrunken; the pulse soft and 90 per minute. He has lost weight considerably. The symptoms indicating diabetes; the urine was tested in presence of the class, and the urinometer indicated 1,030 sp. gr. A specimen was mixed with a solution of sulphate of copper so that a bluish tinge is produced and an excess ot caustic potassa added; heat then gradually changed the mixture to a dark orange red.

The nature of diabetes mellitus is of course still in obscurity, though now traceable beyond the kidneys, where it was formerly located. The starch and gum of the food is converted into sugar before it can enter the blood, and normally the sugar is changed, probably into lactic acid, before entering the arterial circulation. However, if the sugar should not be thus changed, the blood would become charged with sugar, and an excess of sugar in the blood acting as a diuretic is carried out by the kid neys. This stimulation may cause enlargement of the kidney, and the coincident loss of blood constituents may lead to tuberculosis.

The indications for treatment are to provide a diet containing little that can become sugar and to promote digestion. Bran bread, lean meat, butter-milk or sour milk fulfill the first requisite, and though it may seem a poor diet, patients who were lean sometimes get fat on it. To assist digestion, Dr. Jones, of New Orleans, who is one of the most active and laborious workers in the country, prescribed fresh rennet macerated in vinegar. The mixture of rennet, 1 oz., to vinegar, 1 pt., should stand two or three days and then be given in tablespoonful doses after each meal. Patients seldom prepare this properly if it is left to them, and a pharmaceutical preparation known as liquid rennet may be directed. Rennet has done more good in this disease than any other remedy in the Professor's experience. He has found temporary benefit from use of tannic acid; and some recent writer has alledged entire success in the use of this article in doses of from 20 grs. to 30 grs. This patient has taken tannin, 10 grs., and tannate of quinia, 3 grs., three times a day. The warm bath is used every evening in order to relieve kidneys, by increasing the activity of the skin. He was advised to continue these remedies with a fluid drachm of the liquid rennet after each meal.

---

CASE V. ALBUMINURIA.—This man's kidneys are congested and their action almost suppressed; the albumen of the blood is passing out through them. Inflammatory attacks without any structural changes may cause albuminous urine, but when there is no structural damage, the symptoms come on suddenly. In Bright's disease they appear slowly, as in the present instance. The patient's urine is rather dark and turbid, 1,007 in specific gravity. Two or three drops of nitric acid throw down a white precipitate which heat makes more dense. Both these tests must be applied as very alkaline urine retains albumen in solution at any temperature; while after the use of copaiba nitric acid will often produce a white precipitate which is cleared up by heat.

The elimination of water being diminished and the albumen escaping, the patient has become dropsical. Whenever the albumen is reduced to 60 in 1,000 parts of the blood, dropsy is manifested. The retained urea sometimes effects the brain, but in this case it acts upon the heart which beats only 50 times in a minute and with corresponding feebleness. The dropsy is merely a symptom and not yet burdensome; if it becomes excessive, it will be much better to relieve the patient by incisions through the skin than to attempt to remove a smaller amount of fluid by exhausting cathartics and diuretics. This case is doubtless one of granular degeneration of the kidneys.

The remedies most valuable are warm baths, iodide of potassium or sodium to operate on the structure of the kidneys. Theory would be against the use of mercurials as the blood is already aplastic. But in practice the bi-chloride of mercury in doses of 1-40 of a grain in a fluid drachm of tincture of cinchona, given at each meal time, has produced decided benefit in many cases in the early stage. Recently large doses of iodide of potassium, 20 grs., three times a day, have been reported very successful. This patient began with 8 grs., which made him sick at the stomach ; a solution of carbolic acid was given to counteract the sickness, and the dose of the iodide was increased to 10 grs. If it can be borne, the dose will be gradually increased to 20 grs., with the design of causing absorption of the exudation in the structure of the kidneys.

---

CASE VI. PARTIAL HEMIPLEGIA, ETC.—The present patient was in the hospital eight months ago during several weeks. At that time he was complaining of a severe and persistent pain in the head, in the parietal region, and through the base from the occiput to the frontal bone. There was a prominence or tumor on the right side of the frontal and parietal bones, and he suffered from hemiplegia of the left side which came on after the pain. He was much improved by treatment, and though not well, he returned to his home in the north division of this city ; the destruction of the north division has brought him again to the hospital. He has cephalic pains, and his mind is impaired, at times deranged ; the hemiplegia is so far partial that he retains slight control of the left limbs.

The appearances indicate a thickening of the dura mater over the sphenoid and ethmoid bones, the tumefaction of the pericranium being of the same character, and due to the influence of remote syphilis. The pressure at the base and side of the brain explain the paralysis and derangement of intellection. It is evidently important to detect the diseased condition early, before any softening or caries has taken place.

The treatment of course is iodide of sodium, or potassium and bichloride of mercury. When there is pain, as in the present case, an anodyne may be added as in the formula below :

℞ Iodide of Sodium, ℨiij.

Bichloride of Mercury, gr. j.

Fl. Ext. Conium, ℥ss.

Simple Syrup, ℥ss.

Aqua Men. Pip, ℥iij.

M.

*Sig.*—One teaspoonful four times a day.

Under this treatment the pain is generally easier in four or five days, and in ten days gone. Then the dose may be reduced to three times a day for two or three weeks, and to twice a day for three or four months. Tonics will be required, both iron and the bitter barks, or the syrup of the hypophosphites; strychnia, gr. 1-40, and nitric acid, gtts. 1½, are tonics particularly adapted to these cases.

